# DAIR (Debridement, Antibiotics and Implant Retention) less effective in hematogenous total knee arthroplasty infections

**DOI:** 10.1186/s13018-019-1324-5

**Published:** 2019-08-28

**Authors:** Kattalin Iza, Xabier Foruria, Jesús Moreta, Iker Uriarte, Ane Loroño, Urko Aguirre, José Luis Martínez de los Mozos

**Affiliations:** 1 Department of Orthopaedic Surgery and Traumatology, Hospital Galdakao-Usansolo, Barrio Labeaga s/n, 48960 Galdakao, Bizkaia Spain; 2 Research Unit, Hospital Galdakao-Usansolo, Galdakao, Bizkaia Spain

**Keywords:** Debridement and implant retention, DAIR, Infected total knee arthroplasty, Prosthetic joint infection

## Abstract

**Background:**

Debridement and irrigation with prosthetic retention followed by antibiotic therapy (DAIR) is one of the treatments of choice in acute infections after a total knee arthroplasty. However, the success rate varies widely in the literature, depending on several factors such as comorbidities of the patient, duration of infection, and microorganisms involved. The goal of this study was to assess the outcomes of this therapeutic option and to identify possible predictors of the result.

**Methods:**

We retrospectively reviewed cases of acute postoperative (≤ 3 months from index procedure) and acute hematogenous periprosthetic knee infections treated with DAIR at our hospital between 2004 and 2016. Overall, 26 knees were included, with a mean age of 73.4 years. Several variables related to patient characteristics, infection type, and surgery were examined to evaluate their influence on outcome, and functional and radiographic outcome were assessed. The mean follow-up was 41 months. A descriptive analysis was carried out on the collected data, and a univariate analysis was performed with the objective of searching for influential factors in the resolution of the infection using the chi-square nonparametric test in the case of the categorical variables and the Wilcoxon test for the continuous ones. Moreover, univariate cox regression analysis was performed.

**Results:**

The overall success rate was 77% at the last follow-up, recording a significantly greater cure in acute infections (93% acute vs 58% acute hematogenous, *p* = 0.03). The infections in which the *Staphylococcus aureus* was isolated had a significantly lower cure rate, with only 33% of success, compared to 82% of the non-aureus microorganisms (*p* <  0.05).

**Conclusions:**

The present study shows a considerable cure rate in the treatment of acute knee infections through DAIR, although patient comorbidities, type of infection, and causative microorganism should be considered for decision-making.

## Introduction

Infection after total knee arthroplasty (TKA) is one of the most devastating complications and one of the most frequent causes of revision, together with aseptic loosening and instability. It is estimated that its incidence is between 1 and 2% after primary prosthesis and up to 5–6% in revision surgery [1]. The gold standard for treating deep chronic infections is two-stage reimplantation since these infections are very difficult to control with debridement and irrigation with prosthetic retention followed by antibiotic therapy (DAIR) [2, 3]. In case of acute postoperative and acute hematogenous infections of TKA, DAIR is one of the treatments of choice. However, its success rate varies widely in the literature (18–100%) [4–6], depending on the factors of the patient, duration of infection, microorganisms involved, debridement technique, type of antibiotic, and duration of the antibiotherapy. In the current literature, controversy remains as to whether DAIR should be performed for both acute postoperative and acute hematogenous infections, or just in the acute ones, since a substantial number of patients with acute hematogenous infections ultimately experience a relapse of infection after this less-aggressive procedure. [7, 8].

The goal of this study was to assess the clinical and radiological results following this therapeutic option, to identify possible predictors of outcome and to compare the outcomes between hematogenous and acute periprosthetic knee infections.

## Materials and methods

We performed a retrospective study of all the patients diagnosed with an acute postoperative or acute hematogenous periprosthetic joint infection (PJI) after TKA who underwent DAIR at our institution during the period between 2004 and 2016. Patient consent and approval from the institutional research ethics board were obtained. We used the criteria established by the Musculoskeletal Infection Society (MSIS) [9] to diagnose infection. Following the criteria of the Proceedings of the 1st International Consensus Meeting on Periprosthetic Joint Infection [10], we performed DAIR in early postoperative infections that occurred within 3 months of index primary arthroplasty with less than 3 weeks of symptoms or in acute hematogenous infection, which were characterized by an acute onset of symptoms, in a previously well-functioning knee arthroplasty, with less than 3 weeks of symptoms, secondary to another infection. All patients who underwent DAIR for acute post-surgical or acute hematogenous PJI and meet the MSIS criteria for PJI were included. Patients who underwent DAIR who did not meet these definitions, those who underwent reimplantation surgery, those with a mega-prosthesis, and those with missing demographic or surgical data were excluded.

We considered culture-negative PJI, as a culture of a joint aspirate or intraoperative specimens negative for aerobic and anaerobic bacteria, in conjunction with purulence surrounding the prosthesis, acute inflammation on histopathologic examination at the time of surgery, or a sinus tract communicating with the prosthesis, with or without prior use of antimicrobials, following the definition given by Marculescu et al. [11]. The infection was considered eradicated in patients with absence of infectious symptoms, normalization on inflammation markers (CRP and ESR), free of antibiotic therapy, and without the need for prosthetic replacement with a minimum follow-up of 1 year. Failure was defined by the Delphi International Consensus Criteria [12] within 1 year of follow-up: It was considered as a failure of treatment if there was an infection recurrence caused by the same microorganism, if any additional subsequent surgical intervention was performed for infection following DAIR, or if death related to PJI happened. Sociodemographic data of the patients were collected, as well as body mass index (BMI), associated medical comorbidities, type of infection, the American Society of Anesthesiologists (ASA) physical status classification system score, and the Age-Adjusted Charlson Comorbidity Index (ACCI) [13].

All operations were performed at our institution by different surgeons with distinct levels of experience (at least > 5 years) in knee arthroplasty. In all cases, we used the same surgical incision and extensive debridement was carried out, collecting samples for microbiology (at least five) and washing with 9–12 l of normal saline. In all cases, polyethylene was replaced. During surgery, no case of loosening of the components was identified and a drain was inserted in all cases.

Postoperatively, all patients received antibiotic therapy. Empirical antibiotics were started after taking intraoperative samples and adjusted when causative organisms were identified and sensitivities determined. All the treatment was carried out by the Infectious Diseases Department of our institution. The mean duration of antibiotic therapy postoperatively was 12 weeks (range, 4–53 weeks), with priority given to biofilm-active antibiotics such as rifampicin and/or fluoroquinolones.

Clinical results were evaluated using the visual analogue pain scale (VAS), the validated Knee Society Score scale (KSS) in Spanish [14], and the level of ambulation. Likewise, a radiographic analysis was performed to determine possible radiolucency or periprosthetic osteolysis, through the Radiographic Evaluation of the Knee Society [15]. Patients were referred for clinical and radiological assessment at 3 weeks, 3 months, 6 months, 1 year, and every 1 to 2 years thereafter. Also, they were followed up by an infectious disease specialist with experience in the management of orthopedic infections. The minimum follow-up period was 1 year.

During the study period, 26 patients (11 women and 15 men) met the inclusion criteria of the study and were treated at our institution. The mean age of the patients was 73 years (range 57–83), with a mean BMI of 30 kg/m^2^ and an age-adjusted Charlson comorbidity index of 4.3. In 88% of the cases, prosthesis implantation was performed at our institution and the main indication was osteoarthritis. Almost all of the infections were on primary prostheses, except for 2 cases, which were on revision prostheses, due to aseptic loosening. The most common type of implant fixation was hybrid (*n* = 21), followed by cemented (*n* = 5). Fourteen of the patients presented an acute knee prosthesis infection, and 12 patients had an acute hematogenous infection. The mean time from index surgery to DAIR was 22 days (range 12–47 days) in acute infections and 26 months (range 4–96 months) in acute hematogenous ones. The mean duration of symptoms was 15 days (range 3–21 days).

### Statistical analysis

A descriptive analysis was carried out on the collected data in which the categorical variables were shown in the form of frequencies and percentages and the continuous variables in means and standard deviations. A univariate analysis was carried out with the objective of looking for influential factors in the resolution of the infection using the chi-square nonparametric test in the case of the categorical variables and the Wilcoxon test for the continuous ones. Moreover, univariate and multivariate Cox-regression analysis were performed. All effects were considered statistically significant when *p* <  0.05. All analyzes were performed using SAS software, version 9.4.

## Results

The mean follow-up was 3.4 years (1–12 years) with 20 knees (77%) free of infection at the last follow-up. The outcome was successful in the majority of the early postoperative infections (93%) whereas in the hematogenous group, only 58% had resolution of the infection (*p* = 0.03). In the analysis of comorbidities (Table 1), none of them showed to influence the risk of failure, performing neither a univariate nor multivariate Cox-regression model. As a dependent variable, the follow-up time to the event was set up. As independent variables, those with a *p* value < 0.20 were included in the model. Based on the unadjusted results (Table 2), the subsequent variables were considered: side, malignancy, hypertension, thyroid disease, and the presence of the infection caused by streptococcus and polymicrobials. Using the backward procedure, the final multivariate model only included the side of the intervention. There was no difference in outcome based on duration of symptoms. The duration of symptoms for the successfully treated group averaged 15.6 days before DAIR, whereas the failure group averaged 14.8 days (*p* = 0.44). However, we observed a trend towards a higher cure rate when debridement was performed in the first 10 days compared to whether it was performed later (87% vs 60%), although statistical significance was not reached (*p* = 0.1). Microbiological findings are presented in Tables 3 and 4. Regarding the causative microorganisms, overall, in 19 cases, it was identified. The most frequently isolated microorganism was gram-negative bacilli (GNB) (27%), followed by coagulase-negative staphylococcus (CoNS) (23%). The infections in which the *Staphylococcus aureus* was isolated (12%) had a significantly lower cure rate, with only 33% of success, compared to 82% of the non-aureus microorganisms (*p* < 0.05).

**Table 1:** Demographic factors for 26 episodes of prosthetic joint infection (PJI) treated with debridement and retention of prosthesis

Variable	Failure (*n* = 6)	Success (*n* = 20)	*p* value
Age*	69.8 (8.7)	74.5 (6.7)	0.18
Gender			0.66
Male	3 (20)	12 (80)	
Female	3 (27.2)	8 (72.7)	
Side			0.47
Right	4 (28.5)	10 (71.4)	
Left	2 (16.6)	10 (83.3)	
Smoker	1 (50)	1 (50)	0.34
Body mass index*	30.4 (3.4)	30 (2.6)	0.62
Deyo-Charlson Index*	5.1 (2.9)	4.0 (1.4)	0.47
ASA			0.12
I	0 (0)	1 (100)	
II	4 (33.3)	8 (66.6)	
III	1 (8.3)	11 (91.6)	
IV	1 (100)	0 (0)	
Comorbidities			
DM	1 (25)	3 (75)	0.92
Obesity (BMI > 30 kg/m^2^)	3 (25)	9 (75)	0.82
Malignancy	1 (50)	1 (50)	0.34
Hypertension	6 (31.5)	13 (68.4)	0.09
Cardiac disease	2 (66.6)	1 (33.3)	0.05
Chronic pulmonary disease	4 (44.4)	5 (55.5)	0.05
Chronic renal failure	1 (25)	3 (75)	0.92
Vascular disease	1 (25)	3 (75)	0.92
Thyroid disease	0 (0)	1 (100)	0.57
Rheumatoid arthritis	0 (0)	2 (100)	0.42
Immunosuppressive therapy	1 (25)	3 (75)	0.92
Steroid therapy	3 (30)	7 (70)	0.50
Repetitive urine infection	0 (0)	3 (100)	0.31

*n* (%); *media (standard deviation)

**Table 2:** Univariate Cox regression analysis

Variable	HR (95% CI)	*p* value
Age	1.05 (0.97–1.31)	0.24
Gender		
Male	Ref	
Female	1.02 (0.40–2.60)	0.97
Side		
Right	Ref	
Left	1.98 (0.79–4.96)	0.14
Smoker	3 (0.34–26.84)	0.33
Body mass index	0.91 (0.78–1.06)	0.23
Deyo-Charlson Index	0.958 (0.721–01.274)	0.77
ASA		
I	Ref	
II	0.34 (0.04–2.95)	0.33
III	0.61 (0.08–5.02)	0.65
IV	0 (0)	0.99
Comorbidities		
Diabetes mellitus	1.62 (0.45–5.85)	0.46
Obesity (BMI > 30 kg/m^2^)	0.83 (0.33–2.08)	0.70
Malignancy	0.25 (0.03–1.98)	0.19
Hypertension	2.86 (0.90–9.11)	0.07
Cardiac disease	0.70 (0.09–5.37)	0.73
Chronic pulmonary disease	0.63 (0.21–1.92)	0.42
Chronic renal failure	1.66 (0.47–5.94)	0.43
Vascular disease	0.87 (0.25–3.04)	0.83
Thyroid disease	6.25 (0.70–55.92)	0.10
Rheumatoid arthritis	1.24 (0.28–5.54)	0.78
Immunosuppressive therapy	1.59 (0.45–5.65)	0.48
Steroid therapy	0.86 (0.32–2.30)	0.77
Repetitive urine infection	1.70 (0.48–6.06)	0.41

**Table 3:** Microbiological findings

	*n*	Success (*n* = 20)	Failure (*n* = 6)	*p* value
GNB	6	67%	33%	0.60
CoNS	5	60%	40%	0.56
*S. aureus*	3	33%	67%	0.12
Streptococcus	3	100%	0	0.31
Polymicrobial	1	100%	0	0.58
Anaerobe	1	100%	0	0.58
Negative cultures	7	100%	0	0.15

*CoNS* coagulase-negative staphylococci, *GNB* gram-negative bacilli

**Table 4:** *S. aureus* versus non-aureus

	*n*	Success (*n* = 20)	Failure (*n* = 6)	*p* value
*S. aureus*	3	33%	67%	< 0.05
Non-aureus	23	83%	17%	

Regarding the KSS at the end of the follow-up, there were no statistically significant differences between the failures and the success treatment group, with a mean value of 67. There was a tendency of a higher KSS in the failure group, who were treated with revision arthroplasty, with an average of 75, compared to the successfully treated group, with an average of 65 (*p* = 0.5). At the final follow-up, mean pain, measured by VAS score, was 4.2 (range, 1–7). Regarding mobility, 25% of patients were able to walk without help, 54% needed a stick, 17% needed two crutches, while 4% were not able to walk. The radiology at the end of the follow-up did not show any case of loosening, although there were 8 patients (31%) with radiolucencies less than 2 mm in the tibial component (zones 1 and 2).

Overall, 7 local complications were recorded after debridement, including hematoma in 2 and wound dehiscence in one. All of them healed with local wound treatment, and none of them needed second debridement. One systemic complication was registered, which consisted in acute thrombocytopenia due to antibiotherapy. No one required second DAIR, and neither mortality due to infection was registered. In 5 of the 6 cases in which debridement failed, a two-stage revision was performed, while in one case suppressive antibiotherapy was chosen. The eradication rate after the two-stage revision was 100%.

## Discussion

DAIR as a treatment modality for both acute postoperative and acute hematogenous PJI remains a controversial issue, since the success rate varies widely in the literature. The main difficulty in interpreting results lies in the different criteria used to select patients for a DAIR, the classification of PJI used, the antibiotherapy protocol, and the follow-up duration among other factors [6].

In the present study, we have shown that a considerable percentage of patients with an early PJI can be successfully treated with DAIR. However, we found that acute postoperative infections have significantly better results as compared to acute hematogenous ones, and this result is widely supported in previous studies [16–22]. Vilchez et al. [23] demonstrated that hematogenous infection is an independent predictor of failure; in their series, it was targeted that the failure rate in hematogenous and early post-surgical PJI was 59% and 25%, respectively. In addition, Lora-Tamayo et al. [24] found poorer prognoses in hematogenous infections in comparison to acute ones. These results could be supported by the fact that hematogenous infection is usually related to a systemic infectious disease with a higher bacteriemic load; therefore, the response to the DAIR in these circumstances could result in less success. The patient’s immunity is an established factor for infection control. Segawa et al. [25] concluded that the compromised immune status was a major factor associated with treatment failure. In our study, we did not identify any statistically significant hazard ratio determining the relationship between the included factors and treatment failure.

Many investigators have demonstrated that the duration of the symptoms before debridement is a crucial prognostic factor for success of DAIR. Narayanan et al. [26] showed that early DAIR (within 2 weeks since the symptoms started) had higher rates of treatment success (82 vs 50%, *p* = 0.024). In our series, no time-dependent variables were associated with success, but a trend towards a shorter time between appearance of symptoms and debridement was observed among successfully treated patients, although we did not find any statistical difference.

The virulence of the causative microorganism is also an important issue. In many studies, *Staphylococcus aureus* has been shown to be an independent risk factor for failure [17, 20, 27–29]. In our series, when comparing outcomes of *S. aureus* to non-aureus, failure was significantly higher in the *S. aureus* group (33% success vs 82% success respectively, *p* = 0.05). In addition, some studies have concluded that the higher rate of failure is found in infections caused by methicillin-resistant *S. aureus* (MRSA) [24, 30–33]. We could not study this factor since our three infections caused by this microorganism were methicillin-sensitive.

When to stop antimicrobial therapy in patients with DAIR and a favorable clinical course remains an open question. In some studies, favorable outcomes have been reported with only 4 weeks of a “short guideline” therapy [4, 25, 34], unlike the long therapy recommended by other authors [35]. In our series, the choice of antibiotic selection and duration of therapy (mean duration 3 months) was made by the Infectious Diseases Department, and we found that the duration was similar for cured patients and for failures. Thus, it seems that, at least for a substantial proportion of patients, it is not necessary to give prolonged antibiotherapy. Nevertheless, we cannot exclude that a longer duration of therapy could have improved overall results.

Finally, our study shows that a considerable percentage of patients with an early PJI can be successfully treated with DAIR, but as a retrospective observational study, there are some limitations due to its design and also that our series includes a small sample size, attributable to the fact that PJI is relatively uncommon. However, all the patients were treated using the same surgical protocol, in the same center, and selected with the same inclusion criteria defined by MSIS.

## Conclusions

Our study emphasizes a highest risk of failure in acute hematogenous infections treated with DAIR. This procedure is a common surgical modality at our institution and may be a successful treatment in acute infections, but with less probability of success when we are facing hematogenous infections.

## Data Availability

The datasets generated during and/or analyzed during the current study are not publicly available due to the data protection law in Spain but are available from the corresponding author on reasonable request.

## Abbreviations

ACCI: Age-Adjusted Charlson Comorbidity Index
ASA: American Society of Anesthesiologists
BMI: Body mass index
CoNS: Coagulase-negative staphylococcus
CPR: C-reactive protein
DAIR: Debridement antibiotherapy and implant retention
ESR: Erythrocyte sedimentation rate
GNB: Gram-negative bacilli
KSS: Knee Society Score
PJI: Periprosthetic joint infection
TKA: Total Knee Arthroplasty
VAS: Visual analogue pain scale

## References

[1] Pavoni GL, Giabbella M, Falcone M, Scorzolini L, Liberatore M, Carlesimo B (2004). Conservative medical therapy of prosthetic joint infections: retrospective analysis of an 8-year experience. Clin Microbiol Infect.

[2] Deirmengian C, Greenbaum J, Lotke PA, Booth RE, Lonner JH (2003). Limited success with open debridement and retention of components in the treatment of acute Staphylococcus aureus infections after total knee arthroplasty. J Arthroplast.

[3] Hirakawa K, Stulberg BN, Wilde AH, Bauer TW, Secic M (1998). Results of 2-stage reimplantation for infected total knee arthroplasty. J Arthroplast.

[4] Tsukayama DT, Estrada R, Gustilo RB (1996). Infection after total hip arthroplasty: a study of the treatment of one hundred and six infections. J Bone Joint Surg Am.

[5] Zimmerli W, Widmer AF, Blatter M, Frei R, Ochsner PE (1998). Role of rifampin for treatment of orthopedic implant–related staphylococcal infections: a randomized controlled trial. Foreign-Body Infection Study Group. JAMA.

[6] Qasim SN, Swann A, Ashford R (2017). The DAIR (debridement, antibiotics and implant retention) procedure for infected total knee replacement. A literature review. SICOT J.

[7] Mont MA, Waldman B, Banerjee C, Pacheco IH, Hungerford DS (1997). Multiple irrigation, debridement, and retention of components in infected total knee arthroplasty. J Arthroplast.

[8] Morrey BF, Westholm F, Schoifet S, Rand JA, Bryan RS (1989). Long-term results of various treatment options for infected total knee arthroplasty. Clin Orthop Relat Res.

[9] Javad Parvizi MD, Benjamin Zmistowski BS (2011). New definition for periprosthetic joint infection. From the Workgroup of the Musculoskeletal Infection Society. Clin Orthop Relat Res.

[10] Parvizi J, Gehrke T, Chen AF (2013). Proceedings of the International Consensus on Periprosthetic Joint Infection. Bone Joint J.

[11] Marculescu CE, Berbari EF, Hanssen AD (2006). Outcome of prosthetic joint infections treated with debridement and retention of components. Clin Infect Dis.

[12] Diaz-Ledezma C, Higuera CA, Parvizi J (2013). Success after treatment of periprosthetic joint infection: a Delphi-based international multidisciplinary consensus. Clin Orthop Relat Res.

[13] Charlson M, Szatrowski TP, Peterson J, Gold J (1994). Validation of a combined comorbidity index. J Clin Epidemiol.

[14] Ewald FC (1989). The knee society total knee arthroplasty roentgenographic evaluation and scoring system. Clin Orthop Relat Res.

[15] Insall JN, Dorr LD, Scott RD, Scott WN (1989). Rationale of the knee society clinical rating system. Clin Orthop Relat Res.

[16] Silva M, Tharani R, Schmalzried TP (2002). Results of direct exchange or debridement of the infected total knee arthroplasty. Clin Orthop Relat Res.

[17] Gardner J, Gioe TJ, Tatman P (2011). Can this prosthesis be saved?: implant salvage attempts in infected primary total knee arthroplasty. Clin Orthop Relat Res.

[18] Geurts JA, Janssen DM, Kessels AG, Walenkamp GH (2013). Good results in postoperative and hematogenous deep infections of 89 stable total hip and knee replacements with retention of prosthesis and local antibiotics. Acta Orthop.

[19] Kuiper JW, Willink RT, Moojen DJ, van den Bekerom MP, Colen S (2014). Treatment of acute periprosthetic infections with prosthesis retention: review of current concepts. World J Orthop.

[20] Rodríguez D, Pigrau C, Euba G, et al. REIPI Group (Spanish Network for Research in Infectious Disease) (2010) Acute haematogenous prosthetic joint infection: prospective evaluation of medical and surgical management. Clin Microbiol Infect. 16(12):1789–95. 10.1111/j.1469-0691.2010.03157.x 21077986

[21] Theis JC, Gambhir S, White J (2007). Factors affecting implant retention in infected joint replacements. ANZ J Surg.

[22] Tintle SM, Forsberg JA, Potter BK, Islinger RB, Andersen RC (2009). Prosthesis retention, serial debridement, and antibiotic bead use for the treatment of infection following total joint arthroplasty. Orthopedics.

[23] Vilchez F, Martínez-Pastor JC, García-Ramiro S (2011). Efficacy of debridement in hematogenous and early post-surgical prosthetic joint infections. Int J Artif Organs.

[24] Lora-Tamayo J, Murillo O, Iribarren JA (2013). A large multicenter study of methicillin-susceptible and methicillin-resistant Staphylococcus aureus prosthetic joint infections managed with implant retention. Clin Infect Dis.

[25] Segawa H, Tsukayama DT, Kyle RF, Becker DA, Gustillo RB (1999). Infection after total knee arthoplasty. A retrospective study of the treatment of eighty-one infections. J Bone Joint Surg Am.

[26] Narayanan R, Anoushiravani AA, Elbuluk AM, Chen KK, Adler EM, Schwarzkopf R (2018). Irrigation and debridement for early periprosthetic knee infection: is it effective?. J Arthroplast.

[27] Azzam KA, Seeley M, Ghanem E, Austin MS, Purtill JJ, Parvizi J (2010). Irrigation and debridement in the management of prosthetic joint infection: traditional indications revisited. J Arthroplast.

[28] Tornero E, García-Oltra E, García-Ramiro S (2012). Prosthetic joint infections due to Staphylococcus aureus and coagulase-negative staphylococci. Int J Artif Organs.

[29] Cobo J, Miguel LG, Euba G (2011). Early prosthetic joint infection: outcomes with debridement and implant retention followed by antibiotic therapy. Clin Microbiol Infect.

[30] Bradbury T, Fehring TK, Taunton M (2009). The fate of acute methicillin- resistant Staphylococcus aureus periprosthetic knee infections treated by open debridement and retention of components. J Arthroplast.

[31] Ferry T, Uçkay I, Vaudaux P (2010). Risk factors for treatment failure in orthopedic device-related methicillin-resistant Staphylococcus aureus infection. Eur J Clin Microbiol Infect Dis.

[32] Triantafyllopoulos GK, Poultsides LA, Zhang W, Sculco PK, Ma Y, Sculco TP (2014). Periprosthetic knee infections treated with irrigation and debridement: outcomes and preoperative predictive factors. J Arthroplast.

[33] Zürcher-Pfund L, Uçkay I, Legout L, Gamulin A, Vaudaux P, Peter R (2013). Pathogen-driven decision for implant retention in the management of infected total knee prostheses. Int Orthop.

[34] Zimmerli W, Ochsner PE (2003). Management of infection associated with prosthetic joints. Infection.

[35] Byren I, Bejon P, Atkins BL (2009). One hundred and twelve infected arthroplasties treated with DAIR (debridement, antibiotics and implant retention): antibiotic duration and outcome. J Antimicrob Chemother.

